# Computational Fluid Dynamics Modeling of Ventilation and Hen Environment in Cage-Free Egg Facility

**DOI:** 10.3390/ani10061067

**Published:** 2020-06-20

**Authors:** Long Chen, Eileen E. Fabian-Wheeler, John M. Cimbala, Daniel Hofstetter, Paul Patterson

**Affiliations:** 1 Department of Agricultural and Biological Engineering, The Pennsylvania State University, University Park, PA 16802, USA; dwh5212@psu.edu; 2 Department of Mechanical Engineering, The Pennsylvania State University, University Park, PA 16802, USA; jmc6@psu.edu; 3 Department of Animal Science, The Pennsylvania State University, University Park, PA 16802, USA; php1@psu.edu

**Keywords:** CFD model, ventilation, poultry, animal zone, temperature, air velocity, animal welfare, computational fluid dynamics

## Abstract

**Simple Summary:**

The goal of this study was to model and evaluate indoor environment for commercial poultry barns to help builders and egg producers accommodate current transitions to cage-free facilities. By modeling, a one-eighth section of a typical cage-free hen house with 2365 individual hens at full-scale, environmental conditions were assessed in terms of important parameters such as temperature, air speed, and static pressure difference. The simulated ventilation rate for the hen house was set at a desirable cold weather ventilation rate at freezing outside conditions. Contours of interior airflow, temperature, and pressure suggested the indoor conditions were maintained within comfortable ranges, especially important as documented at the bird level. Our findings demonstrated computational fluid dynamics (CFD) modeling is a powerful tool to assess a ventilation system and its impact on the indoor environment for comfort, particularly within the animal-occupied zone of livestock housing.

**Abstract:**

Poultry facilities are going through an evolution in design due to growing demands for cage-free eggs and egg products without unified guidelines to accommodate these transitions. The goal of this study was to help builders and egg producers assess current ventilation design within cage-free production facilities for conditions that impact hen comfort and welfare. The method of evaluation was simulation of the indoor environment of a hen house via computational fluid dynamics (CFD) modeling with individual hens modeled at a typical stocking density. This paper describes the development of a three-dimensional model of a commercial floor-raised cage-free hen house that is cross-ventilated to document current environmental conditions. A one-eighth section of the barn was modeled at full-scale using existing ventilation schemes with each bird represented by a hen-shaped, heated, solid body. A conventional top-wall inlet, side-wall exhaust (TISE) ventilation configuration was modeled for this study. The simulated ventilation rate for the hen house was approximately 3 m^3^/h (1.77 ft^3^/min) per hen resulting in 7092 m^3^/h (4174 ft^3^/min) for the 2365 birds, which falls at the higher end of the desired cold weather (0 °C) ventilation range. Contours of airflow, temperature, and pressure were generated to visualize results. Three two-dimensional planes were created at representative cross-sections to evaluate the contours inside and outside the barn. Five animal-occupied zones within each of the model planes were evaluated for practical hen comfort attributes. The simulation output suggested the TISE standard ventilation system could limit air speed to a comfortable average of 0.26 m/s (51 ft/min) and the temperature could be maintained between 18 and 24 °C on average at the bird level. Additionally, the indoor static pressure difference was very uniform averaging −25 Pascal (0.1 inches of water), which falls in the normal range for a floor-raised hen house with negative-pressure ventilation during cold weather conditions. Findings confirmed that CFD modeling can be a powerful tool for studying ventilation system performance at the bird level, particularly when individual animals are modeled, to assure a comfortable indoor environment for animal welfare in poultry facilities.

## 1. Introduction

In recent years, a shift to cage-free eggs has been prompted by consumer demands and pressure from animal rights’ activists. Several companies [1] have pledged to source 100% of their eggs from cage-free facilities over the next five to 10 years. However, the cage-free egg market still has a long way to go to meet retailer pledges [2,3]. Currently, about 57.2 million layer hens are housed in cage-free environments in the United States, which represents approximately 15 to 17% of the entire US laying hen population [3]. The nation’s cage-free flock needs to expand to 139.5 million birds by 2030 [4] to meet retailer demand, indicating that an additional 43% of the current layers need to be housed cage-free [5].

The lack of unified guidelines and consumer understanding of industry conflict regarding what “cage-free” means has left egg producers in a gray area, unclear about transitioning to cage-free housing, what housing system to switch to, and how to most effectively manage a cage-free facility to maintain optimal production [1]. Common cage-free housing systems include aviary systems, convertible cages, and floor housing. Each housing configuration has its advantages and drawbacks with regard to stocking densities, mortality rates, disease control, and nuisance of eggs laid outside of nest-boxes (a.k.a., floor eggs). In addition, poultry building size has increased dramatically for most new construction, yet ventilation system design for comfort and air movement has not kept pace with recent performance-based, systematic design methods.

Properly designed and managed ventilation systems are vital in poultry production. The importance of uniform indoor environmental conditions in livestock housing has been amply demonstrated [6,7]. When airflow is properly distributed, humidity, particulate matter, and gases are removed. This creates a healthier and more productive environment for animals and for the people who work in livestock buildings [8]. Additionally, airflow patterns decisively govern the uniformity of indoor environmental parameters such as temperature, contaminant levels, and humidity [9]. Therefore, a well-designed ventilation system plays a critical role in providing adequate indoor air quality while properly distributing airflow to meet application needs.

In recent years, the agricultural engineering community has embraced computational fluid dynamics (CFD) to model airflow fields and thereby predict the performance of ventilation systems under various conditions for the sake of comprehensive design and evaluation [10]. Moreover, CFD modeling allows full control of the influencing factors and provides universal data in the computational domain for relatively little time and expense [11]. Hence, researchers have been encouraged to use CFD simulations to design better poultry housing ventilation systems to address indoor environmental problems. Mistriotis and Jong used a two-dimensional CFD model to study a broiler house with natural ventilation and found that employing a solar chimney improved indoor temperature and air velocity [10]. Seo et al. evaluated airflow, indoor air temperature, and the ventilation efficiency of a naturally ventilated broiler house for four modified ventilation systems using CFD simulation [12]. Osorio-Saraz et al. used CFD modeling to predict the emissions of ammonia in an uninsulated broiler house with natural ventilation [13]. All these studies have validated their CFD models with experimental data with good agreement. However, modeling the birds within these buildings was always challenging and required high computational costs and, consequently, to date there has been minimal research on modeling full-scale facilities including animals in the model. Fortunately, CFD simulations of livestock building ventilation systems that include modeling of animal bodies and heat production is evolving as computational power has improved. Seo et al. [14] concluded that the accuracy of CFD models of swine barn ventilation was enhanced if animals were included in the model.

The study reported here documents the performance during cold weather conditions of a ventilation system design used in poultry houses in North America. Indoor air movement, temperature distribution, and static pressure difference between interior and outside were modeled in a three-dimensional CFD simulation. The goal was to document ventilation performance for a cage-free, floor-raised hen house with a particular focus on air speed and temperature for comfortable conditions within the zone occupied by the modeled birds.

## 2. Materials and Methods

### 2.1. Development of the CFD Model

Based on previous investigations [15,16,17,18], the standard *k-ε* turbulence model [19,20] with enhanced wall functions was used for the present work, using the commercial software package FLUENT v19.1 [21]. Simulations were conducted on Penn State’s high-performance computing (supercomputer) resources.

#### 2.1.1. The Study Poultry House

**Figure 1:**
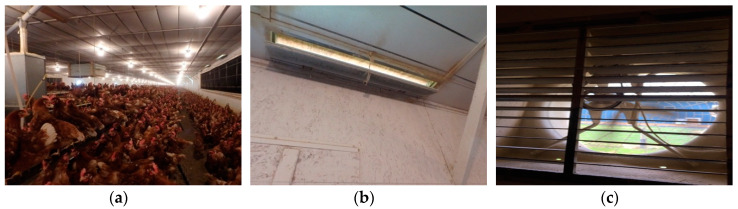
Pictures of the study hen house. (**a**) Interior view, (**b**) close-up view of a ceiling inlet, (**c**) close-up view of a sidewall exhaust fan.

The study poultry house was a floor-raised layer house, located in Lititz, Pennsylvania (Figure 1). The barn had interior dimensions of 162.15 m (532 ft) long and 13.72 m (45 ft) wide. The height of side walls was 2.73 m (8.96 ft) and the thickness was 0.19 m (7.5 in.). The barn had a flat interior ceiling and shallow 4/12 exterior roof slope. The number of birds housed was 19,950 and the stocking density was 1115 cm^2^/bird (1.2 ft^2^/bird). Colony nesting boxes were located at the barn centerline with a total square footage of 232.26 m^2^ (2500 ft^2^) and included 250 compartments (1.67 × 4 ft).

The hen house had a cross-flow ventilation system that included inlets at the top of each sidewall and sidewall exhaust fans. The inlets drew fresh outdoor air from under the building eaves, which is typical in North America, and is referred to in this study as “top-inlet sidewall exhaust” (TISE). There were a total of 84 rectangular ventilation inlets (each 1.17 m (46 in.) by 0.20 m (8 in.)) near the eaves along both sides of the building with hinged baffles that could be adjusted to control the inlet opening area. Four exhaust fans (0.91 m (36 in.) in diameter) were located along one sidewall of the barn and used during brooding, as well as for cold and mild weather. For warm and hot weather, a tunnel ventilation system was employed (not modeled in this study).

#### 2.1.2. Two- and Three-Dimensional Computational Domains

A two-dimensional domain was initially used to study the indoor air conditions for this barn [22]. However, the mechanical ventilation performance of the real hen house was not accurately represented by the two-dimensional simulations because the inlets were not physically located in the same two-dimensional plane as the exhaust fan [15]. Therefore, a three-dimensional model was developed for subsequent work to obtain more accurate and realistic results [23].

The three-dimensional domain of the studied floor-raised house was developed at full-scale for one-eighth of the actual length. A one-eighth building length allowed a representative model size including an exhaust fan and the proportional number of fresh air inlets. A central section of the house was chosen to minimize end-wall effects, and symmetry boundary conditions were applied.

**Figure 2:**
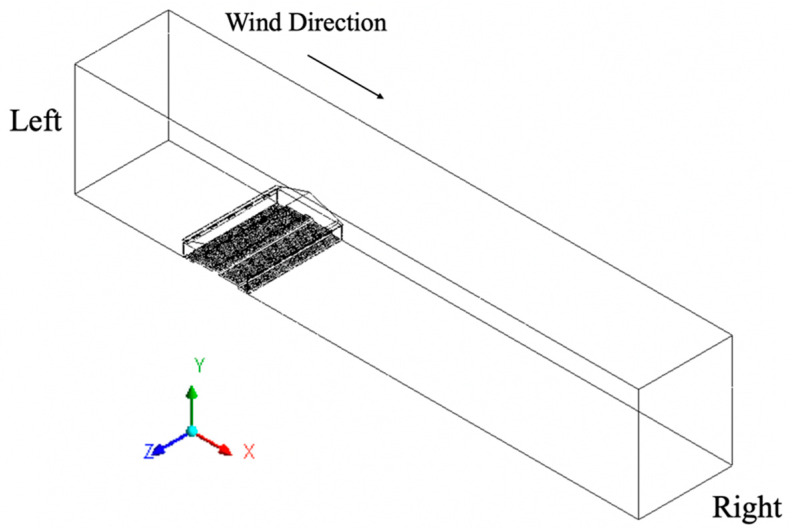
Modeled one-eighth section of the hen house within the computational domain.

The computational domain was much larger than the house itself to properly model airflow inside and outside the hen house (Figure 2). The height of the computational domain was 24.4 m (80 ft) and the width was 128.2 m (420.6 ft). An outdoor wind speed of 2.0 m/s (4.5 mph) was simulated perpendicular to the barn sidewalls, blowing along the x-axis from left to right across the computational domain. Note the distance between the left end of the domain and the house was much shorter than that from the right end to the house because an extended domain far from the target hen house reduced reverse flow conditions at domain boundaries, especially for the downwind side [24].

**Figure 3:**
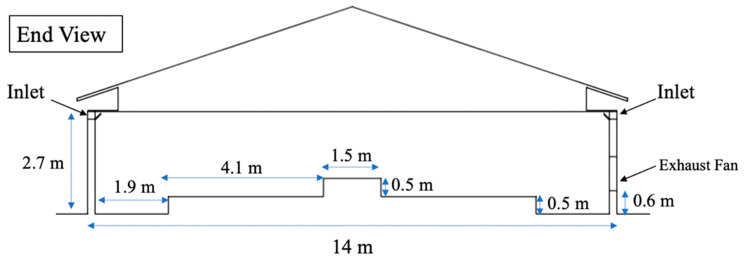
Cross-section of the study hen house with dimensions annotated.

The house section was modeled using dimensions obtained from construction blueprints provided by collaborators. The length of the modeled hen house was 20.3 m (66.5 ft) and the width was 14 m (46.0 ft) including the thickness of walls. The width of the nesting area was 1.5 m (5.0 ft) at the center of the house (Figure 3). Two symmetric slatted floor areas were on both sides of the nesting, each 4.1 m (13.5 ft) wide. Two 1.9-m (6.4 ft) wide litter areas were modeled adjacent to each sidewall. The elevation of the slatted floor and the nesting area were 0.5 m (1.6 ft) and 1.0 m (3.3 ft), respectively. Note, in this study, walls and roof of the hen house were assumed ideally insulated. Thereby, no envelope loads were taken into account in the simulation process.

**Figure 4:**
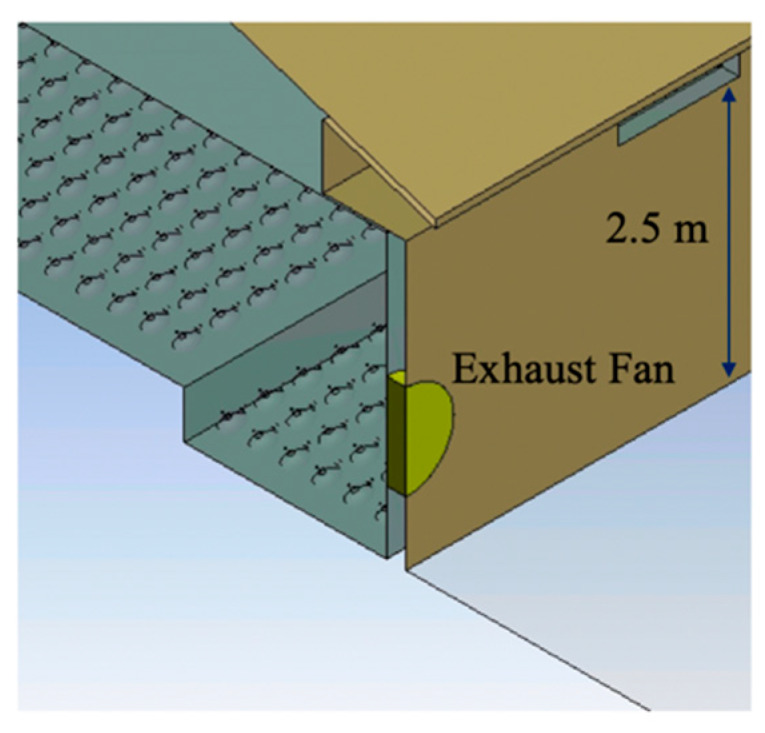
A close-up view of an inlet and fan at the right sidewall in the computational model.

The modeled ventilation scheme included 10 inlets above each sidewall (symmetrical about the building centerline), spaced evenly along the building eaves (representing roughly one-eighth of the total building inlets), and one of the four sidewall exhaust fans located in the right sidewall (Figure 4). Ventilation inlets at the top of both sidewalls were 2.5 m (8.3 ft) above the ground. Note that only half of the exhaust fan was modeled to take full advantage of symmetry while reducing computational needs (Figure 4).

#### 2.1.3. Modeling the Birds

**Figure 5:**
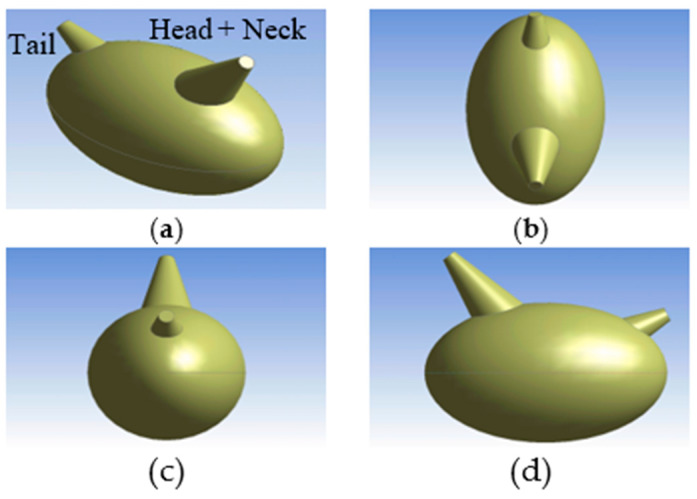
A single hen model with simplified geometry from various views: (**a**) Isometric, (**b**) top, (**c**) back, (**d**) right side.

**Figure 6:**
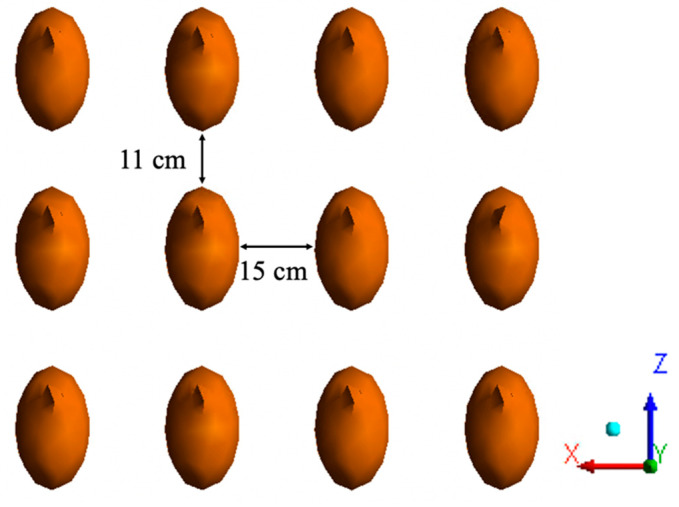
Top view of 12 hen models (evenly distributed).

The simplified geometry of individual hens was represented using a combination of an ellipsoid and two conical projections (Figure 5). The height of each hen model was 0.20 m (0.67 ft), the width was 0.15 m (0.5 ft), and the surface area was 0.11 m^2^ (1.24 ft^2^), which was equivalent to a hen body weight of approximately 1.6 kg (3.5 lb) based on the relationship between body weight (M) and surface area (S) (Equation (1) [25]). In addition, an estimated distance between birds was calculated based on the stocking density by assuming all the birds were evenly distributed; note that this condition was fairly well reflected in Figure 1. In total, 2365 hens were modeled, which is approximately one-eighth of the total birds housed. The distance between the bottom of a single bird and the ground was 7 cm (2.76 in.) [26]. The side-to-side distance between two adjacent birds was 15 cm (6 in.), and the distance from the back of a bird to the front of the neighboring bird was 11 cm (4.4 in.) (Figure 6).

(1) $$M=\sqrt[{0.667}]{S/0.081}$$

### 2.2. Boundary Conditions

Several types of boundary conditions or cell zones were adopted in the CFD simulation inside and outside the hen house.

- Wall: The ground, ceiling, roof, slatted floor, nest boxes, litter area, inlet baffles, sidewalls, animal surfaces, and the top surface of the computational domain were defined as wall boundary conditions. Note that all “walls” were defined as non-slip walls except for the top surface of the computational domain which was defined as a zero-shear stress wall with no resistance along the surface.
- Body heat: Each hen model was defined as a solid volume, whose surface was defined as solid wall with a constant typical hen body temperature of 42 °C (107.6 °F). A heat generation rate of 4467 W/m^3^ was assigned to the outer hen surfaces [23].
- Symmetry: This boundary condition was used where the physical geometry of interest and the expected pattern of the flow/thermal solution had mirror symmetry, which included the front and back surfaces of the computational domain along the *z*-axis and both near and far ends of the house, as those surfaces represented internal faces that accounted for one-eighth of the actual scenario.
- Wind velocity: The left surface of the computational domain was defined as a boundary condition of “velocity inlet” with a specified wind speed of 2.0 m/s (393.7 ft/min) traveling along the positive *x*-axis (Figure 2). Wind velocity profile was constant with elevation.
- Pressure outlet: The right surface of the computational domain was assigned a boundary condition of “pressure outlet” through which flow exits to atmospheric pressure.
- Atmosphere: For this study, the temperature of the atmosphere was specified as 0 °C (32 °F) at zero-gauge pressure.
- Interior: Two faces of each inlet perpendicular to the wind direction were assigned boundary conditions of “interior.” This allowed those “surfaces” to be open faces to represent a portion of the computational domain through which air could flow. Additionally, the exhaust fan had two faces defined as interior through which air would flow.
- Driving force 3D fan zone: The volume of the exhaust fan was defined as a “3D fan zone” where the entire fan volume was considered a fluid cell zone, which simulated the effect of an axial fan by applying a distributed momentum source. In addition, the fan was defined with a hub radius of 5 cm (2 in.), a tip radius of 46 cm (18 in.), and a thickness of 5 cm (2 in.). Rotational speed was specified as 60 radian/s (573 rpm). A constant pressure-jump of 18 Pascal (0.072 inches of water) was applied across all the cells in the fan zone in order to assure the desired hen ventilation rate [24] would fall in a reasonable range for the actual situation (cold weather and hen density). This pressure-drop value along with other inputs of the exhaust fan, comprised the “3D fan zone”, which was set to drive the entire flow regime.

### 2.3. Mesh Settings

The meshing process in this study was performed using ANSYS-Meshing software [20]. Mesh skewness was utilized to assess meshing quality before starting a CFD simulation, and standard mesh convergence studies were performed. The final mesh contained 12.1 million cells and was of adequate quality according to standard practice.

### 2.4. Solver Settings

In this study, steady-state calculations were performed. The mass, momentum, and energy equations were discretized using a second-order scheme, whereas a first-order scheme was applied for the turbulent kinetic energy and turbulent dissipation rate equations.

Various solid materials were modeled, including steel, wood, ash-solids (litter on floor), and layer-body (hen). A designated material was created to represent the surface of the hens with parameters retrieved from appropriate thermal and physiological references [27]. The air was modeled as an incompressible ideal gas.

Monitoring points were defined within the computational domain for observing solution convergence. The convergence criteria were satisfied when both the monitoring variable at these selected points and the residual values were stabilized [28]. The solution for this study was fully converged by 2500 iterations.

### 2.5. Data Visualization

**Figure 7:**
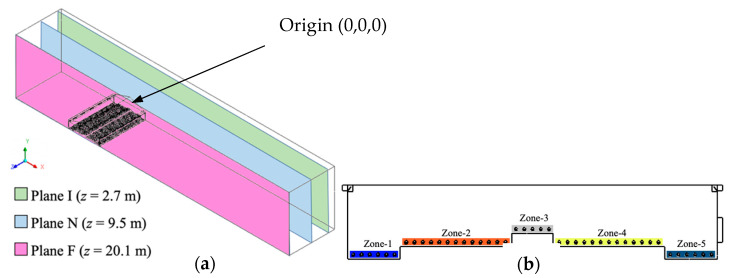
Data visualization. (**a**) Three designated two-dimensional planes based on the ventilation feature within each where “I” stands for “inlet”, “N” stands for “no-ventilation features”, and “F” is short for “exhaust fan”; (**b**) five zones in house cross-section that represent areas at hen level.

Three two-dimensional reference planes were created to analyze the results of the simulations (Figure 7). These parallel cross-sectional slices along the *z*-axis represented locations in the house impacted by ventilation features. Plane I was located at *z* = 2.7 m (9 ft) and contained two inlets. Plane N was located *z* = 9.5 m (31.1 ft) with no ventilation features. Plane F crossed the exhaust fan at a location where *z* = 20.1 m (65.8 ft). Note that all three planes crossed a row of animal models for the purpose of analyzing the performance of ventilation at the animal level. The three selected planes adequately represent all locations in the entire computational domain. In addition, the percentage house floor cross-sectional areas that had inlets, represented by Plane I, or no ventilation features, represented by Plane N, and with a fan (Plane F) were 29%, 69%, and 2%, respectively. Therefore, roughly two-thirds of the house area was dependent on the other third for achieving ventilation system performance.

Five zones were created to characterize the ventilation performance at the animal level (Figure 7). Each zone was 0.25 m (10 in.) tall to represent the height of the hen air space. Zone 1 and Zone 5 (litter area near sidewalls) were symmetric with a width of 1.78 m (5.83 ft). Similarly, Zones 2 and 4 (slatted floor areas with feed and water) were identical at 3.94 m (12.9 ft) wide. Zone 3 represented animals at the middle of house (nest-box area) and was 1.52 m (5 ft) wide. The five zones play an important role in evaluating indoor air conditions from the perspective of the hens within the three two-dimensional planes.

### 2.6. Ventilation Rate

The ventilation rate during cold weather required to maintain good air quality for a single 1.6 kg (3.5 lb) hen should be approximately 0.59 to 2.97 m^3^/h/hen (0.35 and 1.75 ft^3^/min [cfm]/hen) [29]. The simulation used a rate of 2.99 m^3^/h per hen (1.77 cfm/hen), on the high end of recommended cold weather ventilation rate, for evaluation at 0 °C. This resulted in a total ventilation rate of about 7092 m^3^/h (4174 cfm) for the study hen house section with 2365 hens. All inlets modeled in this study had 3.81-cm (1.5 in.) tall openings to provide a suitable static pressure difference [30] at this ventilation rate.

### 2.7. Evaluation Criteria

All simulation data exported for analysis were assessed according to specific criteria to determine whether the ventilation system provided suitable indoor conditions for hens. The desired temperature range was between 18 and 24 °C (64 to 75 °F) with an indoor air speed at hen level between 0.25 and 1.0 m/s (49 to 197 ft/min). Additionally, the normal range of static pressure difference between indoor and outdoor conditions would be 10 to 25 Pascal (0.04 to 0.10 inches of water gage) for this type of negative pressure ventilation system. The cold, fresh air entering through the inlets at a high velocity was expected to travel along the ceiling toward the middle of the house while mixing with warm air already in the building before dropping into the animal-occupied area.

### 2.8. Statistical Analysis

We needed to quantitatively compare simulation data of important environmental parameters from five different animal-occupied zones on each reference plane. For a steady-state analysis, there was only one data point at a given location in the domain and it did not vary with time after convergence. Therefore, exported data points in each zone were treated as repeated measurements captured throughout that animal zone. The simulation data were fit to a mixed-effects model as shown in Equation (2):

(2) $$y_{ijk}=\mu +\tau_{i}+\beta_{j}+{\left(\tau \beta \right)}_{ij}+\varepsilon_{ijk}\;i=1,\;\ldots ,\;a_{E};j=1,\;\ldots ,\;b_{E};k=1,\;\ldots ,\;c_{E}\;\varepsilon_{ijk}\;i.i.d.\;N\left(0,\sigma^{2}\right)$$

where μ was the mean of a parameter (air speed, temperature, pressure), $\tau_{i}$ was the effect of the ith plane, $\beta_{j}$ was the effect of the jth zone, ${\left(\tau \beta \right)}_{ij}$ was the interactive effect of plane and zone, and $\varepsilon_{ijk}$ was the random error for the kth data point that was independent and identically distributed (i.i.d)). Herein, the values $a_{E}$ and $b_{E}$ represented the number of levels of factors: Reference plane and zone, which equaled 3 and 5, respectively. The value of $c_{E}$ referred to the total number of data points exported for analysis. The primary intent was to verify whether changing planes and zones had effects on the results of parameters of interest. From a statistical perspective, such comparison made sense only when the null hypotheses were rejected for the following overall significance test:

1. *H*_0_: There was no difference in the means of factor *Plane.*
2. *H*_0_: There was no difference in the means of factor *Zone.*
3. *H*_0_: There was no interaction between factors *Plane* and *Zone.*

The hypotheses were tested using analysis of variance (ANOVA) using R Studio v1.2 [31]. The *p*-values were compared to the sensible significance factor α=0.05 to determine whether the $H_{0}$ would be rejected. Tukey’s multiple comparison procedure was used [32] to compare means from different planes and zones of interests with a family-wise confidence level of 95%. Three ANOVA procedures, corresponding to the data sets for the three parameters of air speed, temperature, and static pressure, are presented in Results.

## 3. Results

Simulation results for the three environmental parameters were visualized at three reference planes by making contour plots for each parameter. In addition, simulation data at the hen level were compared quantitatively for five different zones in each plane to investigate the suitability of indoor air conditions.

### 3.1. Air Velocity Analysis

**Figure 8:**
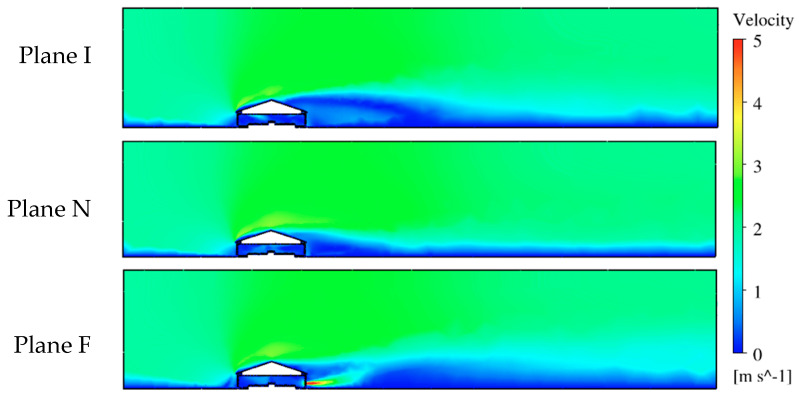
The contour of air velocity magnitudes at three planes with a legend of colors ranging from 0 to 5 m/s. Plane I with inlets, Plane N with no ventilation features, and Plane F with exhaust fan.

**Figure 9:**
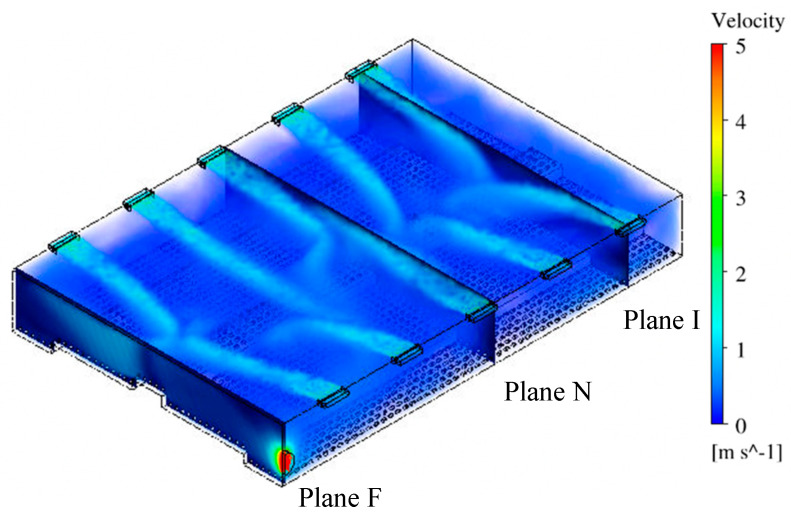
Isometric view of house showing the location of three selected planes and overall airflow patterns and air velocity magnitudes inside the hen house with inlets at the top of both sidewalls and sidewall exhaust fan. Plane I with inlets, Plane N with no ventilation features, and Plane F with exhaust fan.

**Figure 10:**
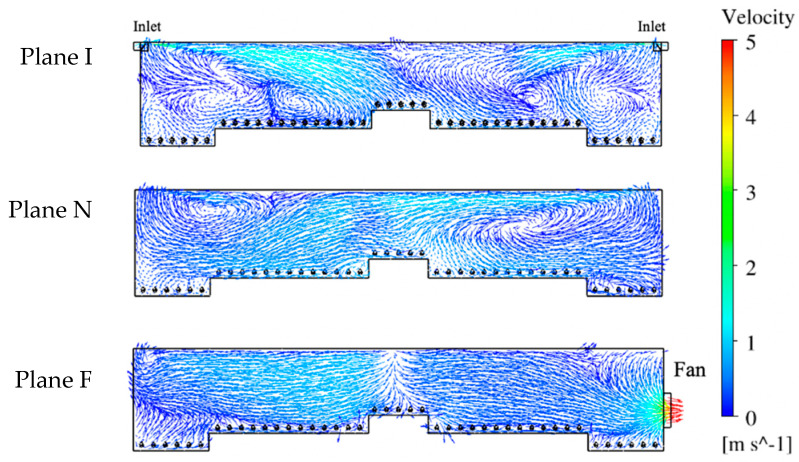
Indoor air velocity vectors at three planes with corresponding ventilation features (inlets and fan). Plane I with inlets, Plane N with no ventilation features, and Plane F with exhaust fan.

Contours of air velocity magnitude (Figure 8 and Figure 9) and velocity vectors (Figure 10) were created to visualize airflow patterns outside and inside the hen house. The house blocked the 2 m/s wind (Figure 8, from left side of the domain). Therefore, an area with lower air speeds (some were zero) was formed at the downwind side of the hen house. However, a high-speed air jet exhausted from the building at the fan location in Plane F. Also, an area at the top of the house was observed with a higher air speed of 3 to 4 m/s (591 to 787 ft/min) for all three planes (yellow-chartreuse area shown in Figure 8). Although the air speed changes could be seen inside the house while looking at the whole computational domain, detailed views of indoor airflow patterns were necessary to evaluate indoor conditions at the three planes (Figure 9 and Figure 10).

Maximum air speed averaging around 6.1 m/s (1200 ft/min) was observed for the 10 inlets within the hen house section (Figure 9). Fast incoming air jets were observed from both inlets at Plane I, as indicated by plotting velocity vectors (Figure 10). The air speed decreased gradually when approaching the center of house where it was impacted by the air jet from the opposing inlet. The upwind inlet introduced air that maintained higher speeds into, and in some cases past, the middle of the house. Throw to the middle of the house is highly desired for air mixing throughout the building [30]. Although the air pattern from the downwind inlet did not throw as far as the upwind inlet, expected air circulation patterns were observed throughout the house, particularly at areas close to both sidewalls. Lower speeds were observed at the bird level, except for the location near the central nest-boxes, which had some faster moving fresh air from the inlets. Additionally, some tiny arrows indicated that the directions of the velocity vectors were not always contained within the reference planes. They might have been oblique or even perpendicular to the plane, indicating lateral airflow, which was reasonable and consistent with actual observations in real poultry house environments.

A similar plot of velocity vectors was created to visualize indoor airflow patterns at Plane N that contained no ventilation features. Although no incoming air jets existed at this plane, strong air movement was observed throughout the cross-section (Figure 10). Airflows around 1.5 m/s (295 ft/min) moved from the upper right to the lower left of the house. In addition, two large air circulation zones formed at both sides of the house. The one at the left was close to the ceiling, and the other was above the birds between the right sidewall and the nest-boxes. The patterns shown on this plane indicated sufficient airflow mixing at suitable velocity.

The maximum air speed at Plane F was 7.6 m/s (1496 ft/min) at the fan discharge (although all values over 5 m/s (984 ft/min) appear red on the color scale (Figure 10)). The air moved from the middle of the house towards the fan within the right side and its speed increased gradually within the fan influence area. However, a strong horizontal air movement can also be seen towards the left sidewall in that portion of the house. Interestingly, the air speeds in the left side of the house were higher than that of the right side despite the fan’s influence. Moreover, the air movement trajectory shown at this plane was almost symmetric, starting from the middle and moving to both sides. Simulations confirm the importance of inlets in determining airflow patterns within the house, even at locations not near an exhaust fan.

### 3.2. Temperature Analysis

**Figure 11:**
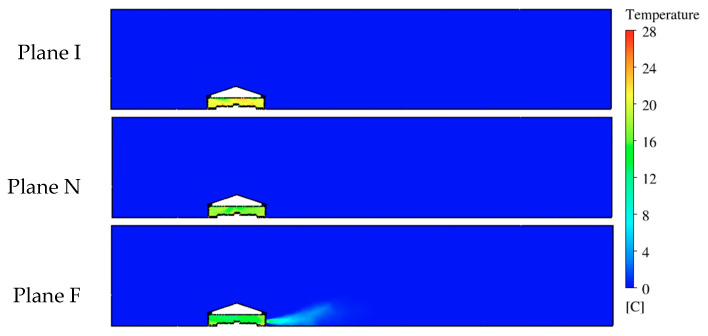
The contour of temperatures at three planes. Plane I with inlets, Plane N with no ventilation features, and Plane F with exhaust fan.

Temperature was another essential parameter for evaluating the performance of this ventilation system. Therefore, temperature contours were created to visualize the distribution of temperatures at each reference plane (Figure 11). The legend shows a scale of color representing the temperature range from the designated outdoor temperature of 0 °C (32 °F) to desirable indoor temperature of 28 °C (82.4 °F). The indoor temperature was much higher than the outdoor temperature due to the addition of bird body heat. The heat from the birds was mixed throughout the house but also showed some areas where more heat was maintained. Warm air exhausted by the fan can be seen as a light blue air jet in Plane F.

**Figure 12:**
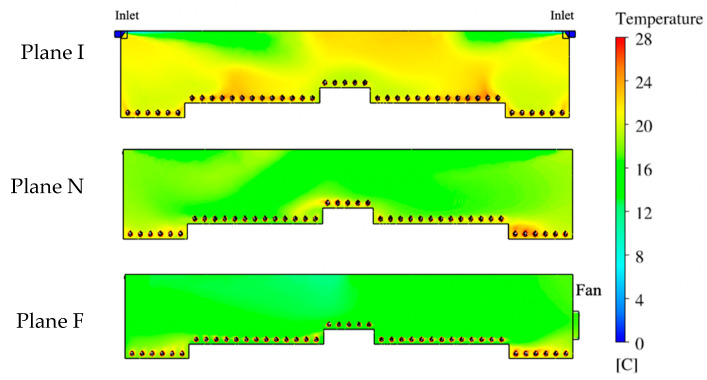
Indoor temperature contours at three planes. Plane I with inlets, Plane N with no ventilation features, Plane F with exhaust fan.

At Plane I, the incoming ventilation air (dark blue) had an initial temperature of 0 °C (32 °F) at the inlet, quickly changing to warmer green and yellow on the temperature-color scale after mixing with indoor air (Figure 12). Warm color-temperatures were found at the animal level and along the ceiling at the middle of the plane. The indoor temperature distribution at Plane N appeared green color (12–16 °C, 53.6–60.8 °F) close to the ceiling and at bird level on the left side between the sidewall and the nest-boxes. The average temperatures inside the house were 21.3 °C (70.3 °F) and 18.9 °C (66 °F) for Planes I and N, respectively.

The temperature contour at Plane F shows large areas of relatively low temperature (10–16 °C, 50-60.8 °F) with the majority of the house appearing green on the color-temperature scale (Figure 12). Warmer air temperatures were observed at the area close to the sidewall, especially on the right side of the house. The average temperature was 16.9 °C (62.4 °F) for this slice of the house. The temperature contour plot illustrates that Plane F had the most uniform indoor temperature distribution of the three reference planes, however, the average temperature was the lowest among the three planes.

### 3.3. Pressure Analysis

Static pressure differences were visualized by creating contour plots at the three reference planes. The atmospheric pressure was set at 0 Pa. Thus, the indoor pressure was negative per design of the negative-pressure exhaust ventilation system. The pressure-color of the inlets at Plane I reflected the outdoor atmosphere. The indoor pressure at Plane I was quite uniform with an average pressure of −24.3 Pascal (Pa). Similar values of indoor pressure were also observed from the contours at Plane N, averaging −24.2 Pa. The indoor pressure at Plane F was again quite uniform, except near the exhaust fan where pressure increased drastically close to the fan. The region at the fan suggested the magnitude of static pressure was as low as −56 Pa.

### 3.4. Animal Zone Analysis

Evaluating indoor air conditions at the animal level was of great importance utilizing the animal-occupied zones (Figure 7). Although Zone pairs 1 and 5 and 2 and 4 were symmetric, the flow properties were not necessarily heterogeneous due to variation in airflow patterns caused by the non-symmetric inlet and fan locations.

The statistical analysis suggested that for all parameters, the effects of plane, animal zone, and the interaction of plane and zone were statistically significant (Table 1). The corresponding results of the three ANOVA procedures, corresponding to data sets achieved from the simulation, are summarized in Table 1. For each parameter, the *p*-value corresponding to each factor was so small that the null hypothesis was rejected at any sensible significance.

**Figure 13:**
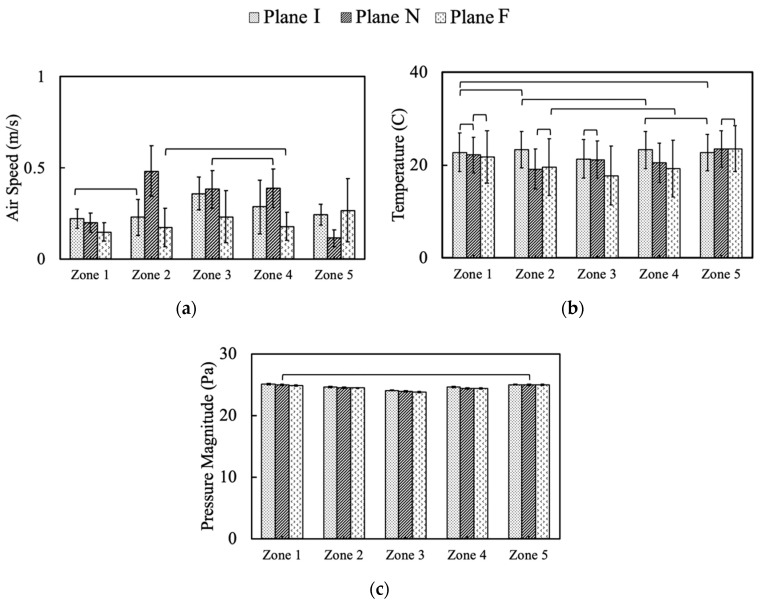
Simulation outputs of environment parameters from five zones at three planes: (**a**) Air speed, (**b**) temperature, (**c**) pressure. Error bars indicate standard deviations while cross bars indicate no statistically significant differences at 95% family-wise confidence level. Plane I with inlets, Plane N with no ventilation features, Plane F with exhaust fan.

The average air speed varied among zones and different planes, as illustrated in Figure 13. Air speeds were within the criteria for hen comfort, e.g., below 1 m/s (~200 ft/min) for draft-free conditions. Some air speeds were considered almost “still air” at 0.25 m/s (50 ft/min), which is desirable during cold weather to provide some air movement but not chill the birds. Zone 3 (nest boxes) was observed to have the highest average air speeds (0.36 m/s) at Plane I (inlets), compared to the other four zones; likely the result of incoming air jets reaching this portion of the bird-occupied area [13]. Zone 1 and Zone 2 at Plane I had quite similar, and the lowest, average air speeds with no statistically significant difference. Average air speed for all five zones within Plane I was 0.27 m/s (53 ft/min) with the standard deviation of 0.13 m/s (26 ft/min). At Plane N (no ventilation features), Zone 2 was observed with the highest mean air speeds around 0.48 m/s, while Zone 5 was the lowest (0.11 m/s) (22 ft/min) with second highest average air speeds at Zone 3 and Zone 4. Average air speed in Plane N at bird level was 0.31 m/s (61 ft/min), and the standard deviation was 0.19 m/s. Within Plane F (exhaust fan) the highest average air speed was found in Zone 5, which may be explained by the influence of air moving toward the exhaust fan. In addition, a large standard deviation was observed with the air speed data of Zone 5 for the same reason. Plane F air speeds from Zone 2 and Zone 4 had no statistically significant differences. Air speed from Zone 3 was slightly lower than that of Zone 5. Zone 1 had the lowest average air speed at Plane F around 0.15 m/s. Average air speed in Plane F at bird level was 0.20 m/s (39 ft/min) with the standard deviation of 0.12 m/s (24 ft/min). The overall air speed, including all zones in all planes, was around 0.26 m/s (51 ft/min) at the bird level, and the overall standard deviation was 0.16 m/s (32 ft/min).

The temperature pattern of each zone was affected strongly by airflow patterns within each plane since the hen heat flux was uniform throughout those zones. Analyses demonstrated some temperature differences had no statistical significance (Figure 13). The average temperature at Zone 3 (center of house) was the lowest (21.3 °C) at Plane I, while the average temperatures of the other four zones were not statistically different from each other (Figure 13). Zone 2 was observed with the lowest temperatures on average at Plane N, while the highest value, 23.5 °C, was found in Zone 5 as the warmed air inside the house moved toward the exhaust fan. Plane F had the lowest temperature compared to other planes in each of the other zones. Temperatures of Zone 1 were the most consistent among the three planes (as was uniformity of air velocity). The average temperature was about 21.5 °C at the hen level for all five zones and three planes of analysis, falling in the bird-comfort range.

Indoor static pressure averages at animal level at all five zones and three planes were quite uniform (Figure 13) with small standard deviations. Although the statistical analysis suggested the difference between each comparison was statistically significant, the magnitude of difference was small. In general, the indoor static pressure ranged was −24 to −26 Pa over all five zones at three planes, which falls in the normal range of static pressure difference for a negative pressure agricultural ventilation system [30]. There appeared to be a trend for slightly higher pressure along the side zones (1 and 5) than in the middle of the house.

### 3.5. Verification and Validation

In this research, verification was completed through a mesh convergence study with strict convergence criteria employed to ensure the meshing quality and the reliability of the CFD model. Unfortunately, funding for this study did not include on-farm validation. Field validation, in this case, would not be a trivial undertaking of time, procuring many and varied instruments, travel, and personnel. However, the simulation results appeared reasonable from a practical standpoint. Airflow patterns (such as large counter-rotating recirculation zones), inlet air speed (6.1 m/s (1200 fpm)), inlet air entrainment and throw (to middle of hen house) reproduce conditions seen in poultry buildings during our previous (non-CFD) investigations [29,30]. Those investigations documented temperature and velocity conditions representing airflow and/or visualization of airflow using smoke. Therefore, the simulation results agreed with the anticipated airflow values and patterns, admittedly “in concept,” with what has been observed in real poultry houses.

## 4. Discussion

The performance of a standard ventilation scheme in a real, commercial, floor-raised, cage-free hen house was modeled with the goal of analyzing the indoor conditions encountered by the birds. The CFD simulation was set up for the top-inlet, sidewall exhaust model (TISE) to provide a ventilation rate of 7092 m^3^/h (4174 ft^3^/min) for the study hen house, which met the demands for 2365 hens during minimum ventilation in cold weather [29]. This ventilation rate was on the higher end of recommended air exchange range for hens during cold weather (3.0 m^3^/h or 1.77 ft^3^/min per hen), which was appropriate for the evaluation at 0 °C. The egg-laying flock was modeled as heated, hen-shaped individuals evenly distributed throughout the building [25,26], which is an improvement over using a heated cube to represent each group of caged hens, as in [24].

Air circulations were observed by velocity vectors throughout the house at three cross-sectional planes (Figure 10). Each vector velocity was indicated by color and direction by length. There was evidence of turbulent and lateral flow differences among the planes of analysis. Plane I showed the most turbulence with the inlet air jet causing strong circulations not only in the anticipated large half-house, countercurrent middle-to-sidewall patterns [7,30] but also several smaller eddies with lateral flow (short arrows) along the house z-axis. Lateral flow makes sense as incoming air needed to distribute through the house section to eventually reach exhaust. Plane I turbulence provided eddies that circulated through the birds collecting body heat brought up into the overall cross-section creating warmer conditions that one might expect in a plane with cold incoming air. It was reassuring that soon after entering the house the incoming freezing air was mixed to a warm temperature with house air in the expanding inlet air jet. This is an intentional design feature of these inlet systems [30] to entrain warm house air with the incoming cold air while it is directed at high velocity to throw across the top of the enclosure. Hence, air that finally drops into the bird-occupied zone is tempered to a comfortable speed and temperature. It appeared that turbulent eddies of the inlet planes incorporated air from other planes. In contrast, Plane F had virtually no lateral flow with the two large air currents moving primarily within that fan-occupied plane from the middle of the house toward each sidewall. Within the plane with no ventilation features, Plane N, turbulent eddies with lateral flow were present. Most of the lateral flow occurred in the lower half of the Planes I and N cross-sections, often taking bird heat along with the airflow. It is notable that the inlet air jet dynamics set in motion the large countercurrent air circulations that were able to dominate airflow patterns throughout the whole one-eighth section of the hen house, even in sections without inlets. Airflow patterns in the house were similar to conditions observed through practical experience of the author team and monitored in other studies of real poultry houses [12,13,26]. Data from the five animal zones showed the average air speed was suitable–around 0.26 m/s (51 ft/min) at the bird level for all three evaluation cross-sectional planes [30].

Temperature distributions inside the house were analyzed by analyzing color contours. The temperature uniformity of the majority of the hen house was excellent with an average value about 21.5 °C at the hen level. This value fell within the range of 18 to 24 °C, which is in the comfort range for birds even on a freezing day outside, with ventilation rate on the high-end of the recommended minimum ventilation range [29,30]. We anticipated that the Plane F could have the highest overall temperature as heated air eventually had to make its way to the exhaust fan. It was slightly warmer near the fan with nearby animal Zone 5 the warmest. It appeared that abundant air eddies in Planes I and N that circulated through the hen zones moved heated air up into those cross-sections more readily than the more uniform, large airflow patterns simulated in Plane F.

Of particular value was documenting simulation conditions at the bird level to ensure the hen environment experience was within comfort criteria, and simulated airflow patterns along with other air conditions were consistent with practical knowledge and previous studies [7,13,26]. Computer simulation results demonstrated impact of the hens and their heat production, as well as the influence of ventilation features on uniformity of indoor air conditions. Temperature was the most uniform inside animal zones (most pairwise comparisons with no statistical significance), which is important for bird comfort and uniform productivity. Because Plane I contained incoming air jets, it tended to have the highest average air speeds compared to that of Planes N and F. Although no ventilation features were included at Plane N, zones in the middle of the house had the highest average air speeds, indicating that air circulation at this plane was influenced by the inlet air jets. Zone 5 had the largest air speed at Plane F due to the influence from the nearby fan. Similarly, the temperature distributions at each plane were influenced by the airflow pattern, e.g., lower temperatures were found where cold inlet air was mixing with warmed room air.

## 5. Conclusions

The analysis presented here demonstrated the value of documenting conditions within the animal-occupied area to obtain localized conditions experienced by the hens that impact their comfort, productivity, and welfare. Overall, the ventilation system within this simulation provided comfortable conditions for the floor-raised hens in terms of temperature and velocity in their animal-occupied zone. Simulated airflow patterns in the house were similar to practical observations and other studies of real poultry houses. Static pressure difference of the hen house was uniform with an average about −25 Pascal, implying this type of negative-pressure ventilation scheme should be functioning well.

Future CFD modeling based on this work can be a powerful tool to analyze ventilation performance and the indoor microclimate. Using realistic dimensions, including modeling individual animals, is expected to improve the accuracy of the CFD simulation. The three-dimensional model presented here can investigate ventilation options. Modifications to the building features and housed hens can assess more complicated ventilation schemes for various types of cage-free poultry houses. In summary, making full use of CFD modeling enables investigators to solve practical problems related to animal housing.

## Figures and Tables

**Table 1:** Analysis of variance (ANOVA) of air speed, air temperature, and pressure from five animal zones at three planes.

Parameter	Factor	df	Pr (>F)
Air speed	Plane	2	<2.2 × 10^−16^
	Zone	4	<2.2 × 10^−16^
	Plane × Zone	8	<2.2 × 10^−16^
	Residuals	25,856	
Temperature	Plane	2	<2.2 × 10^−16^
	Zone	4	<2.2 × 10^−16^
	Plane × Zone	8	<2.2 × 10^−16^
	Residuals	25,856	
Pressure	Plane	2	<2.2 × 10^−16^
	Zone	4	<2.2 × 10^−16^
	Plane x Zone	8	<2.2 × 10^−16^
	Residuals	25,856	

## References

[1] A look at Cage-Free Housing Solutions & Practices for a Changing Market. https://www.val-co.com/modern-hen-look-cage-free-housing-solutions.

[2] Cage-Free Commitments by Companies. https://welfarecommitments.com/cage-free/.

[3] Retailers’ Cage-Free Pledges Demand Millions of Layers. https://www.wattagnet.com/articles/26618-retailers-cage-free-pledges-demand-millions-of-layers.

[4] Monthly USDA Cage-Free Shell Egg Report. https://www.ams.usda.gov/mnreports/pymcagefree.pdf.

[5] U.S. Flock Trends and Projections Report. https://www.eggindustrycenter.org/browse/files/031214b1b8ad4315a5320838596139df/view.

[6] Gebremedhin K.G., Wu B. (2005). Simulation of flow field of a ventilated and occupied animal space with different inlet and outlet conditions. J. Therm. Biol..

[7] Norton T., Sun D.W., Grant J., Fallon R., Dodd V. (2007). Applications of computational fluid dynamics (CFD) in the modelling and design of ventilation systems in the agricultural industry: A review. Bioresour. Technol..

[8] Spoolder H.A.M., Edwards S.A., Armsby A.W., Corning S. A Within Farm Comparison of Three Different Housing Systems for Finishing Pigs. Proceedings of the First International Conference on Swine Housing.

[9] Boulard T., Kittas C., Roy J.C., Wang S. (2002). SE-Structures and Environment: Convective and ventilation transfers in greenhouses, Part 2: Determination of the distributed greenhouse climate. Biosyst. Eng..

[10] Mistriotis A., De Jong T. (1997). Computational Fluid Dynamics (CFD) as a tool for the analysis of ventilation and indoor microclimate in agricultural buildings. Neth. J. Agric. Sci..

[11] Li H., Rong L., Zhang G. (2017). Reliability of turbulence models and mesh types for CFD simulations of a mechanically ventilated pig house containing animals. Biosyst. Eng..

[12] Seo I.H., Lee I.B., Moon O.K., Kim H.T., Hwang H.S., Hong S.W., Bitog J.P., Yoo J.I., Kwon K.S., Kim Y.H. (2009). Improvement of the ventilation system of a naturally ventilated broiler house in the cold season using computational simulations. Biosyst. Eng..

[13] Osorio-Saraz J.A., Olivera Rocha K.S., de Ferreira Tinôco I.F., Gates R.S., Barreto Mendes L., Zapata O.L., Damasceno F.A. Use of CFD modeling for determination of ammonia emission in non-insulated poultry houses with natural ventilation. Proceedings of the ASABE Annual International Meeting.

[14] Seo I.H., Lee I.B., Moon O.K., Hong S.W., Hwang S.W., Bitog J.P., Kwon K.S., Ye Z., Lee J.W. (2012). Modelling of internal environmental conditions in a full-scale commercial pig house containing animals. Biosyst. Eng..

[15] Worley M.S., Manbeck H.B. (1995). Modeling particle transport and airflow in ceiling inlet ventilation systems. Am. Soc. Agric. Eng..

[16] Harral B.B., Boon C.R. (1997). Comparison of predicted and measured airflow patterns in a mechanically ventilated livestock building without animals. J. Agric. Eng. Res..

[17] Rong L., Bjerg B., Zhang G. (2015). Assessment of modeling slatted floor as porous medium for prediction of ammonia emissions—Scaled pig barns. Comput. Electron. Agric..

[18] Osorio-Saraz J.A., Tinôco I.F.F., Rocha K.S.O., Mendes L.B., Norton T. (2016). A CFD based approach for determination of ammonia concentration profile and flux from poultry houses with natural ventilation. Rev. Fac. Nac. Agron..

[19] Launder B.E., Spalding D.B. (1974). The numerical computation of turbulent flows. Comput. Methods Appl. Mech. Eng..

[20] Cengel Y.A., Cimbala J.M. (2007). Fluid Mechanic: Fundamental and Applications.

[21] ANSYS (2009). User Guide.

[22] Fabian-Wheeler E., Chen L., Hofstetter D., Patterson P., Cimbala J. Modeling Hen House Ventilation Options for Cage-Free Environment and Welfare Improvements. Proceedings of the 10th International Livestock Environment Symposium (1st U. S. Precision Livestock Farming Symposium).

[23] Chen L., Fabian-Wheeler E., Hofstetter D. Ventilation Options Modeling for Laying Hens in Cage-Free Environment: Three-Dimensional Case. Proceedings of the ASABE 2018 Annual International Meeting.

[24] Pawar S.R., Cimbala J.M., Wheeler E.F., Lindberg D.V. (2007). Analysis of poultry house ventilation using computational fluid dynamics. Trans. ASAE.

[25] Walsberg G.E. (1978). The relationship of the external surface area of birds to skin surface area and body mass. J. Exp. Biol..

[26] Cheng Q., Wu W., Li H., Zhang G., Li B. (2018). CFD study of the influence of laying hen geometry, distribution and weight on airflow resistance. Comput. Electron. Agric..

[27] Mutaf S., Kahraman N.S., Firat M.Z. (2008). Surface wetting and its effect on body and surface temperatures of domestic laying hens at different thermal conditions. Poult. Sci..

[28] Li H., Rong L., Zhang G. (2016). Study on convective heat transfer from pig models by CFD in a virtual wind tunnel. Comput. Electron. Agric..

[29] Fabian-Wheeler E.E. (2017). Personal Communication.

[30] Inlets for Mechanical Ventilation Systems in Animal Housing. https://extension.psu.edu/inlets-for-mechanical-ventilation-systems-in-animal-housing.

[31] Venables W.N., Ripley B.D. (2002). Modern Applied Statistics with S.

[32] Kutner M.H., Nachtsheim C.J., Neter J., Li W. (2004). Applied Linear Statistical Models.

